# Preparation and Characterization of a High-Performance Foam Extinguishing Agent with Sulfobetaine and Polyoxyethylene Ether for Solid Fires

**DOI:** 10.3390/polym17192579

**Published:** 2025-09-24

**Authors:** Huizhong Ma, Liang Cheng, Lan Zhang, Liyang Ma, Jia Deng, Ao Zhao, Xin Jiang, Fei Wang

**Affiliations:** School of Mechanics and Safety Engineering, Zhengzhou University, Zhengzhou 450001, China; iehzma@zzu.edu.cn (H.M.); cl398273994@163.com (L.C.); ielzhang@zzu.edu.cn (L.Z.); mly2020@zzu.edu.cn (L.M.); dengjialucky@zzu.edu.cn (J.D.); zhaoao2829@163.com (A.Z.); jiangx0909@163.com (X.J.)

**Keywords:** foam wettability, foam adhesiveness, microscopic observation, fire suppression experiments

## Abstract

Although extensive studies have been conducted on the component ratios and performance of fire extinguishing foams, most research has not explored the coupling relationship between foam wettability and adhesion. Therefore, this study aims to develop an efficient foam extinguishing agent for solid fires by focusing on both wettability and adhesion. First, the influence of chemical functional groups on foam wettability and adhesion was elucidated, and the contributions of individual components to foam properties were experimentally investigated. Second, adhesion and wettability tests revealed a negative correlation between these two properties, consistent with variations in foam solution viscosity and wetting time. Third, a novel adhesion evaluation method was proposed, defined as the time required for foam to flow a fixed distance on inclined wooden surfaces; longer flow times indicated stronger adhesion. Fourth, foaming and fire suppression experiments confirmed the practical performance of the optimized formulations. A composition containing 8 wt% Polyoxyethylene ether and 5 wt% Sulfobetaine yielded a wetting-type foam suitable for rapid cooling, whereas 8 wt% Polyoxyethylene ether combined with 9 wt% Sulfobetaine produced an adhesive-type foam capable of persistent attachment to combustibles. Microscopic observations further demonstrated that foams with superior extinguishing performance developed dense lamellae.

## 1. Introduction

Fire remains a major public safety concern, posing ongoing risks to human life, property, and the environment. Current fire suppression technologies commonly employed in buildings include water sprinklers, gaseous agent, dry chemical powders, and conventional foam systems [1,2]. Water sprinklers are cost-effective and offer broad coverage, but they may cause damage to equipment and are ineffective against electrical fires. Gaseous suppression systems can rapidly control flames through oxygen displacement, yet high concentrations of heptafluoropropane pose anesthetic risks to occupants, while CO_2_ may lead to oxygen deprivation and asphyxiation. Dry chemical agents are suitable for a wide range of fire types, but their residues can corrode sensitive equipment and are difficult to clean. Traditional water-based foams can form oxygen barriers, yet they suffer from poor foam stability, limited resistance to reignition, and often rely on fluorinated surfactants, which raise concerns over environmental persistence and potential toxicity. Due to certain limitations in the efficiency, environmental performance, and adaptability of traditional fire extinguishing agents to various scenarios, the development of an A-class foam specifically suited to the demands of modern architectural environments has become an urgent and crucial area of research.

In order to develop fire extinguishing foams with excellent firefighting performance, low cost, and environmental friendliness, scholars both domestically and internationally have conducted extensive research on foam material design, firefighting mechanisms, performance optimization, and practical application verification. These studies have provided valuable insights into exploring foam formulations, determining research directions, and identifying the limitations of current research.

Many researchers have investigated the impact of various additives or factors on foam performance and have successfully developed foams with excellent properties. These studies have significantly influenced the formulation of foams in this work.

For example, Wang et al. investigated the effect of nonionic silicone surfactants on AES [3]. However, they also observed a reduction in foam height. Li et al. investigated the synergistic interactions in binary mixtures of tea saponin (TS) and AES [4]. Li et al. discovered the interaction between anionic surfactant (SDS) and salts [5]. Zhang et al. explored the application of alkylamide propyl betaine in foam systems [6]. Li et al. demonstrated that the addition of urea can lower the critical micelle concentration, improve foam stability, and reduce fire-extinguishing time [7]. Dong et al. studied the influence of metal ions on SDS-based foam systems and found that metal ions inhibit water diffusion in the foam lamella, thereby enhancing stability [8]. Sheng et al. formulated a mixed foam system consisting of short-chain fluorocarbon surfactant (FC-50), anionic hydrocarbon surfactant (SDS), hydrophilic SiO_2_ nanoparticles, and water-soluble polymers such as sodium carboxymethyl cellulose (CMC) and xanthan gum (XG) [9].Qiu et al. prepared high-performance foaming agents using SDS and nonionic surfactant alkyl polyglucoside (APG), with hydrolyzed polyacrylamide (HPAM) as a foam stabilizer [10]. Dinesh et al. developed a foam combining SDS and citric acid (CA), exhibiting excellent mechanical strength and antioxidant performance [11]. Liu et al. developed a highly stable gel foam system using SDS, HPAM, and organic chromium (SD-107), which exhibited excellent temperature and salt resistance as well as sealing efficiency [12].

Building on the research and insights provided by numerous scholars, this study identifies sodium alkyl sulfate (SAS) as a crucial surfactant that plays a vital role in foam formulations. Therefore, sodium alkyl sulfate (SAS) is employed as the primary foaming agent to develop a foam with superior performance. In contrast to the formulations proposed by previous studies, this formulation includes the addition of a wetting agent, enabling the foam to maintain a stable structure while also exhibiting enhanced wetting properties. Based on this optimized formulation, the foam’s performance is thoroughly validated through adhesion and wettability tests, microscopic analysis, and fire suppression experiments.

Many researchers have conducted studies on the preparation and fire-extinguishing performance of foams. For example, Yang et al. investigated a fluorocarbon cationic–hydrocarbon anionic surfactant foam [13]. Zhang et al. studied an alkylamide propyl betaine foam [6]. Wang et al. examined a fluorocarbon surfactant foam containing imidazole [14]. Lou et al. investigated both a 6% AFFF foam extinguishing agent and a 6% PF foam extinguishing agent [15]. Jia et al. studied a novel aqueous film-forming foam extinguishing agent [16]. Yang et al. further investigated a hydrocarbon–perfluorinated branched short-chain fluorocarbon surfactant foam [17]. Wang et al. examined an imidazolium short-chain fluorocarbon surfactant foam [18]. These studies primarily focused on foam surface tension, expansion ratio, and fire-extinguishing time. The comparative results are summarized in Table 1.

From the comparative data in Table 1, the foams developed in this work exhibit superior performance across multiple aspects. Both the wetting-type foam and the adhesive-type foam show relatively low surface tension, a property that promotes rapid spreading and effective wetting of solid fuel surfaces. The expansion ratios (8.2 and 7.8) are moderate but sufficient for practical fire suppression. Although lower than those of AFFF (25.75) and PF foam (17.78), excessively high expansion ratio are often unfavorable due to rapid drainage and reduced stability. Most notably, the fire-extinguishing times of the wetting-type foam (12 s) and adhesive-type foam (20 s) are substantially shorter than those of other reported foams, underscoring their high efficiency in interrupting combustion and cooling the fuel surface.

Many scholars have conducted research on the wettability and adhesion of foams, which has greatly contributed to the direction of this study. For instance, Geng et al. investigated the impact of different wettability particle combinations on foam stability, finding that a combination of two wettability particles can perform better than a single wettability particle [19]. Wang et al. discovered that modified hydrophilic SiO_2_ nanoparticles significantly improve foam wettability [20]. Gholipour et al. analyzed the adsorption behavior of anionic, nonionic, and zwitterionic surfactants on silica nanoparticles, exploring the role of surfactants in altering nanoparticle surface wettability and enhancing foam stability [21]. Eknapaku et al. carefully examined the wettability of foam nickel (NF) using X-ray photoelectron spectroscopy (XPS) and water contact angle (WCA) measurements [22]. Ajayi et al. studied the improvement of foam thermal performance and wettability in epoxy-based foam composite materials (EBFC) [23]. Besson et al. explored the rheology of three-dimensional aqueous foams and the relationship between adjacent bubble adhesion [24]. He et al. investigated the rheological properties and adhesion characteristics of foamed CRA binders [25]. Dai et al. researched the adhesion behavior of compressed silicone foam under different compression ratios and surface roughness [26].

Although substantial research has been conducted on the wettability and adhesion properties of foams, relatively few studies have explored the coupling relationship between these two characteristics. This paper introduces a novel, straightforward method for quantitatively assessing foam adhesion, providing a macroscale perspective on this property, and investigates its correlation with the inclination angle and drop height. Furthermore, the study examines the coupling behavior of adhesion and wettability in both foam solutions and the foams themselves. Through a series of controlled wettability and adhesion experiments, complemented by microscopic analysis of the functional groups in foam stabilizers and wetting agents, this research reveals a strong interrelation between the foam solution properties and the resulting foam characteristics. Specifically, it identifies a negative feedback effect between foam adhesion and wettability, demonstrating their mutually inhibitory relationship. This work underscores the complex interplay between these two foam properties and provides a deeper understanding of their interdependence in foam performance.

Despite the valuable contributions of this study, it is acknowledged that there are certain limitations. While numerous scholars have investigated the fire suppression mechanisms and performance characterization of foam, this study has primarily focused on macroscale experiments and the analysis of functional group properties. For instance, Nie et al. utilized both experimental methods and molecular dynamics simulations to screen four surfactants, examining their wettability and adsorption capacities [27]. Zhang et al. explored the interactions between Polyoxyethylene Alkyl Ethers (AEO) and Sodium Dodecyl Sulfate (SDS) through molecular dynamics simulations [28]. Xu et al. proposed that the fire suppression mechanism of water film-forming foam is largely attributed to its superior cooling, covering, and smothering effects [29]. Dlugogorsk et al. examined the compatibility of water film-forming foams with seawater [30]. Ping et al. analyzed the variation in foam lifetime [31]. Yan et al. identified that the foam extinguishing mechanism primarily involves cooling, covering, and smothering actions [32]. Consequently, this study, while providing insights into the macroscopic properties of foam, could benefit from further advancements. Future work that integrates molecular dynamics simulations would allow for a more detailed examination of the interaction processes of functional groups, thus providing a deeper understanding of the fundamental mechanisms governing foam behavior and performance.

In this study, Sodium Alkyl Sulfate (SAS) was used as the primary foaming agent, Alcohol Ethoxylate Sulfate (AES) as the auxiliary foaming agent, Betaine surfactants (Sulfobetaine) as the foam stabilizer, Polyoxyethylene ether surfactants (Polyoxyethylene ether) as the wetting agent, and Butyl Glycol as the cosolvent, to prepare a high-performance Class A foam. In terms of the scientific novelty, this study innovatively explored the negative feedback relationship between the wettability and adhesion of foam solutions through functional group analysis, and designed a novel experimental setup to test foam adhesion. The relationship between foam adhesion and inclination angle was also investigated. Foam performance was validated through foaming, fire extinguishing experiments, and microscopic observations. The foam designed in this study not only demonstrates excellent performance but also fulfills the dual requirements of effective thermal protection and adequate wettability and adhesion. In terms of benefits to science, the wetting-type and adhesive-type foams demonstrated superior fire-extinguishing performance, with extinguishing times of 12 s and 20 s, respectively, effectively meeting diverse fire suppression requirements. In terms of the justification of the adopted standards, all experiments conformed to GB 27897-2011, China’s mandatory standard for Class A foam extinguishing agents, ensuring standardized and reproducible results [33].

## 2. Materials and Methods

### 2.1. Materials

This study utilized five primary reagents. All these reagents are sourced from Chinese companies. Sodium Alkyl Sulfate (SAS, 99%) was purchased from Tianjin Yongda Chemical Reagent Co., Ltd, Tianjin, China. Alcohol ethoxylate sulfate (AES, 70%), Betaine surfactants (Sulfobetaine, 35%), and Polyoxyethylene ether surfactants (Polyoxyethylene ether, 99%) were purchased from Linyi Lusen Chemical Co., Ltd, Linyi, China. Butyl Glycol (Butyl Glycol, 99%) was purchased from Zhengzhou Pioneer Chemical Reagent Factory, Zhengzhou, China

### 2.2. Foam Solution Preparation

**Figure 1:**
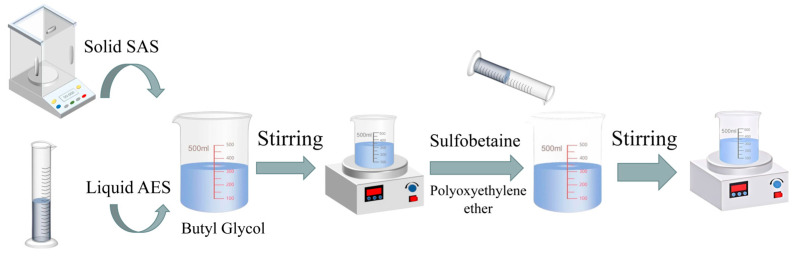
Foam configuration.

In this study, an orthogonal experimental design was employed to prepare 25 different foam concentrate formulations by varying the ratios of foam stabilizers and wetting agents, as shown in Table 2. The foam solutions prepared in this study were concentrated foam stock solutions, which were diluted with water during experiments. As shown in Figure 1, to prepare the solution, a measured amount of the primary foaming agent, auxiliary foaming agent, and deionized water were first weighed and mixed thoroughly in a beaker. The mixture was stirred for 30 min. Subsequently, the foam stabilizer, wetting agent, and the remaining deionized water were slowly added to the beaker and stirred for an additional 30 min until the solution was clear and free of precipitates. The resulting concentrate was stored under cool, shaded conditions.

### 2.3. Key Physical Property Testing of Foam Solution

The GB 27897-2011 standard, which specifies the requirements for Class A foam extinguishing agents, is the current mandatory standard in China. It defines the terminology, product classification, performance requirements, test methods, inspection rules, labeling, packaging, transportation, and storage for Class A foam extinguishing agents. This standard applies to the design, production, and inspection of Class A foam extinguishing agents. All performance evaluations of foam solutions in this study were conducted with reference to the provisions outlined in GB 27897-2011.

#### 2.3.1. Viscosity Measurement of Foam Solutions

According to the viscosity measurement procedure for foam solutions outlined in the Chinese national standard GB 27897-2011, the apparent viscosity of the foam solutions was determined using a DV-1 digital display rotational viscometer (Shanghai Pingxuan Scientific Instruments Co., Ltd., Shanghai, China). Since foam solutions exhibit non-Newtonian behavior, their apparent viscosity varies with shear rate; therefore, measurements were conducted at a constant rotational speed. Given the relatively low viscosity of the solutions, Rotor No. 0 was selected, and a rotational speed of 60 rpm was maintained throughout the tests. All experiments were performed under standard laboratory conditions. Each measurement was repeated three times, and the mean value was reported with two decimal places [34,35].

#### 2.3.2. Wettability Test of Foam Solutions

According to the wetting performance measurement procedure for foam solutions outlined in the Chinese national standard GB 27897-2011, the wettability of the foam solutions was determined using cotton fabric (Shanghai Juhong Instrument & Equipment Co., Ltd., Shanghai, China) disks. Prior to testing, the cotton disks were preconditioned overnight under controlled temperature conditions, and the test solutions were prepared. During the experiment, the immersion clamp was first rinsed with a small amount of the test foam solution, and then adjusted to hold the cotton disk in a nearly vertical orientation. Timing was initiated when the cotton disk just contacted the liquid surface and the clamp was released, and stopped when the disk began to sink automatically. Each foam solution was measured in three consecutive trials, and the arithmetic mean of the three measurements was reported as the wetting time, with results retained to two decimal places.

#### 2.3.3. Surface Tension Measurement of Foam Solutions

According to the standard specified in GB 27897-2011, the surface tension of the foam in the blended system was measured using a QBZY-3 fully automatic surface tensiometer (Shanghai Fangrui Instruments Co., Ltd., Shanghai, China). When the platinum plate was immersed into the test solution, it experienced a downward force due to the liquid’s surface tension, which acted to pull the plate as far into the liquid as possible. Once the liquid’s surface tension and other related forces reached equilibrium with the counteracting forces, the immersion of the plate ceased. At this point, the instrument’s balance sensor measured the immersion depth and converted it into the corresponding surface tension value of the liquid. Each foam solution was measured in three consecutive trials, and the mean surface tension value was reported with two decimal places.

#### 2.3.4. Foam Expansion Ratio Test

The foam expansion ratio of the blended system was determined using the compressed air jet method, in accordance with the standard specified in GB 27897-2011. In this study, the foam expansion ratio was tested using the drainage method [36]. Due to the high experimental consumption, the substantial variation in expansion ratios among different formulations, and the limited relevance to the study’s focus on wettability and adhesion, repeated measurements were not performed. The calculated values are reported with one decimal place. Before the experiment, the system pressure was adjusted to an appropriate level and the initial weight of the foam measuring device (*m*_1_) was recorded. The compression system was then activated, the pressure valve was opened, and foam was injected. The foam was collected using a foam collector (Zhejiang Runlan Technology Co., Ltd., Zhejiang, China), and the weight of the collected foam (*m*_2_) was recorded. The foam expansion ratio (*F*) was calculated using the following formula:

*F* = *ρV*/(*m*_2_ − *m*_1_) (1)

In the formula, *F* represents the expansion ratio; *ρ* represents the density of the foam solution, with the unit of grams per milliliter (g/mL). Take *ρ* = 1.0 g/mL; *V* is the volume of the foam receiver (mL), *m*_1_ is the initial weight of the foam measuring device (g), and *m*_2_ is the weight of the foam measuring device after being filled with foam (g).

### 2.4. Key Foam Physical Property Parameter Testing

In this study, the foam solution was foamed, and its surface tension, wettability, viscosity, and adhesion properties were measured and characterized.

#### 2.4.1. Foam Wetting Performance Test

In this study, the method for testing the wettability of the solution was improved. Prior to the experiment, the equipment was treated in a suitable environment, and the foam concentrate was mixed with deionized water in a specified ratio to prepare the solution, which was then foamed in a beaker. During the experiment, a dry cotton cloth was weighed and immersed in the beaker. After 1 min, the cotton cloth was removed, excess foam was skimmed off using a glass rod, and the cotton cloth was weighed again using a balance. The difference in weight before and after immersion was used to measure the foam’s wettability. For each foam formulation, the change in wetting mass of the cotton fabric was measured in triplicate, and the results are reported to two decimal places.

#### 2.4.2. Foam Viscosity Test

The viscosity of the foam was measured using the DHR-2 rotational rheometer, manufactured by TA Instruments, Waters Corporation, Milford, MA, USA. Before the experiment, self-calibration and initialization were performed, and the instrument was adjusted to the appropriate height. An appropriate amount of foam sample was taken using a glass cup for testing. The viscosity of each foam formulation was measured in triplicate, and the results are reported to two decimal places.

#### 2.4.3. Foam Adhesion Test

1. Vertical Angle Foam Adhesion Test

A wooden board with dimensions of 50 cm in length, 15 cm in width, and 0.5 cm in thickness was used for the adhesion test. Scales were marked at 5 cm, 10 cm, 15 cm, 20 cm, and 25 cm along the long side of the board, and a fixed foam release position was selected at the top of the board to eliminate the influence of surface roughness variations. During the test, the surface of the board was first wetted to eliminate any liquid traces that could affect the accuracy of the experiment. Then, 2 mL of foam was measured and dispensed using a pipette from the fixed position at the top of the board. Timing was started, and the time taken for the foam to flow past each test position was recorded. The adhesion of each foam formulation was tested in triplicate, and the results are reported to two decimal places.

2. Inclined Angle Foam Adhesion Test

**Figure 2:**
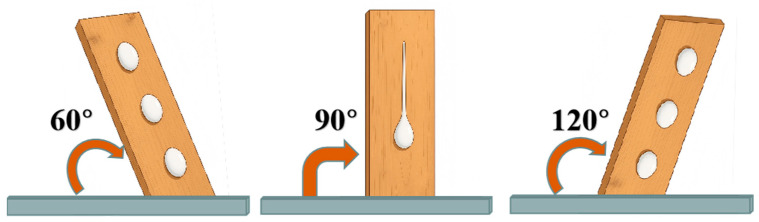
Schematic diagram of foam adhesion test.

In preliminary experiments, positive inclination angles of 30°, 45°, and 60°, as well as negative inclination angles of 120°, 135°, and 150°, were tested. It was found that due to the foam’s good adhesion and relatively high viscosity, the foam did not flow down the board at lower angles. Therefore, this study selected foam adhesion tests at 60° and 120° inclination angles, as shown in Figure 2.

The same wooden board used in the vertical angle test was employed to eliminate any potential board-related effects. During the test, the board was placed horizontally on the constructed test stand, and a fixed foam release position was selected at the top of the board. The angle of the stand was kept fixed during the experiment. The surface of the board was wetted, 2 mL of foam was measured and dispensed using a pipette from the fixed position at the top of the inclined board, and timing was started. The time taken for the foam to flow to the bottom of the board was recorded. The experiment was repeated three times, and the average value was calculated.

### 2.5. Microscopic and Fire Extinguishing Experiments

#### 2.5.1. Foam Fire Extinguishing Performance Test

The A-class fire extinguishing test was conducted using a Yellow Pine Wood Stack of 2A specification (Zhangjiagang Guangxing Wood Industry Co., Ltd., Zhangjiagang, China), in compliance with national standards. The experiment involved igniting the wood stack with an oil pan and allowing it to burn freely. When the mass of the wood stack decreased to 53–57% of its original weight, the oil pan was removed, signaling the end of the preignition phase and the beginning of fire extinguishing [37]. Extinguishing was carried out using a fire extinguisher. The time from the start of fire extinguishing to the complete extinction of the visible flames, and the time at which the smoke produced by the wood stack combustion dissipated, were recorded as the extinguishing time. After the fire was extinguished, the wood stack was observed for ten minutes to check for any reignition. Due to the high consumption and cost associated with the fire suppression experiments, and the considerable variation in extinguishing times among different formulations, each test was conducted only once, with results reported to one decimal place.

#### 2.5.2. Foam Microscopic Observation

In this study, foam morphology was observed using a Leica DM750 microscope, manufactured by Leica Microsystems, Wetzlar, Germany. During the experiment, the microscope was adjusted with the eyepiece magnification set to 10× and the objective lens magnification set to 4×, achieving a total magnification of 40×. Foam samples were dipped for testing, and the light source and focal point were adjusted to achieve the clearest foam images. The foam states at 0, 1, and 5 min were recorded using a camera to observe changes in the foam over time. Due to the significant variability in foam behavior at the microscopic level across different formulations, observations were conducted only once.

## 3. Results and Discussion

### 3.1. Introduction to Experimental Components

#### 3.1.1. Introduction to Polyoxyethylene Ether

**Figure 3:**
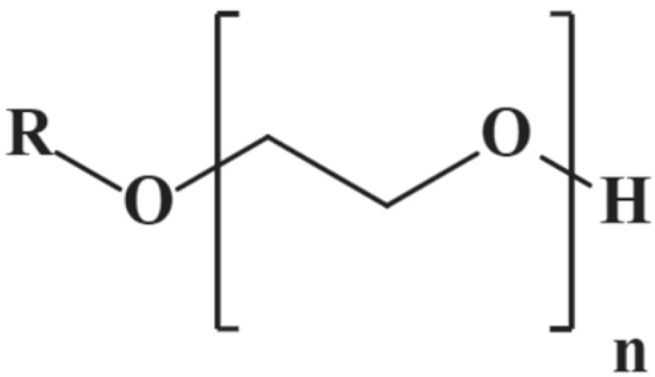
Schematic diagram of Polyoxyethylene ether molecule.

As shown in Figure 3, Polyoxyethylene ether is a nonionicsurfactant synthesized via the addition polymerization of fatty alcohols and ethylene oxide. It is commonly represented by the general formula RO(CH_2_CH_2_O)_n_H, where R denotes the alkyl chain of the fatty alcohol, and *n* indicates the number of ethylene oxide (EO) units. The molecule features a hydrophobic alkyl chain at one end and a hydrophilic polyoxyethylene chain at the other. Polyoxyethylene ether contains four primary functional groups: The alkyl chain (R) acts as the hydrophobic tail, which drives micelle formation in aqueous environments. The ether bond (–O–) connects the fatty alcohol moiety to the EO chain, contributing to the flexibility of the molecular structure. The polyether segment [(–CH_2_CH_2_O)_n_–] serves as a hydrophilic group, determining the solubility and emulsifying capability of the molecule. The hydroxyl group (–OH) at the terminal end ensures sufficient hydrophilicity, enhancing water solubility and gel-forming ability.

#### 3.1.2. Introduction to Sulfobetaine

**Figure 4:**
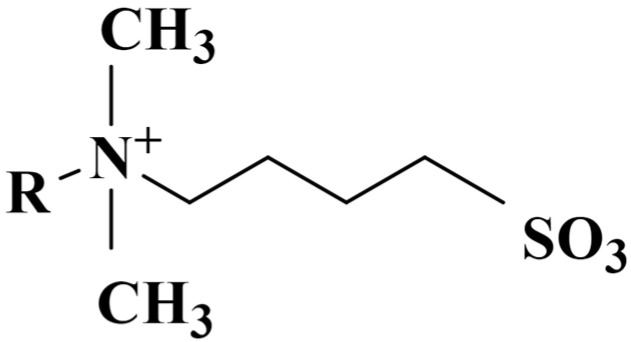
Schematic diagram of Sulfobetaine molecule.

As shown in Figure 4, Sulfobetaine contains three primary functional groups: The alkyl chain (R–) functions as the hydrophobic moiety, promoting molecular adsorption at the gas–liquid interface and facilitating micelle formation, thereby enhancing the compactness of the foam film and regulating interfacial tension. The quaternary ammonium group (–N^+^(CH_3_)_2_–) constitutes the hydrophilic core of the molecule, bearing a permanent positive charge and maintaining stability across a broad pH range; it effectively modulates interfacial electrostatics and mitigates repulsive forces between foam films. The sulfonate group (–SO_3_^−^), a strongly hydrophilic anionic moiety, forms an internal salt with the quaternary ammonium cation, conferring exceptional salt tolerance and electrolyte stability to the foam system, thereby ensuring consistent foam performance even under conditions of high salinity or hard water.

### 3.2. Analysis of Foam Solution Properties

#### 3.2.1. Analysis of Viscosity Testing of Foam Solutions

**Figure 5:**
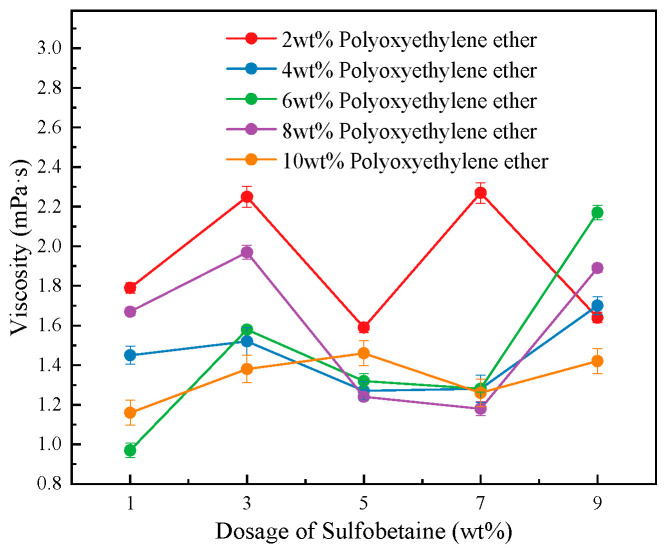
The viscosity of foam solution with the dosage of foam stabilizer Sulfobetaine.

As shown in Figure 5, when the concentrations of the primary foaming agent, secondary foaming agent, and cosolvent are kept constant, the trend of foam solution viscosity is analyzed with varying concentrations of the foam stabilizer Sulfobetaine under different concentrations of the wetting agent Polyoxyethylene ether. It can be observed that, regardless of the Polyoxyethylene ether concentration level, the viscosity of the foam solution increases initially with the addition of Sulfobetaine, then decreases, and subsequently increases again, reaching an optimal viscosity performance when the Sulfobetaine concentration is 3 wt%. Interestingly, at lower concentrations of Polyoxyethylene ether, the foam solution exhibits a higher maximum viscosity. Furthermore, under low Sulfobetaine concentrations (1 and 3 wt%), the viscosity is significantly affected by the Polyoxyethylene ether concentration, whereas under higher Sulfobetaine concentrations (5, 7, 9 wt%), the viscosity values across different Polyoxyethylene ether levels are more concentrated. This suggests that Sulfobetaine demonstrates better compatibility with Polyoxyethylene ether at lower concentrations.

From a microscopic perspective, Polyoxyethylene ether, as a nonionic surfactant, forms hydrogen bonds between its polyoxyethylene chains and water molecules, which promotes the formation of solution structures and enhances intermolecular connectivity, resulting in increased solution viscosity. Meanwhile, Sulfobetaine’s zwitterionic structure partially dissociates in water, generating electrostatic repulsion and forming micelles, both of which contribute to viscosity elevation. The combination of the two surfactants produces a synergistic effect. Due to the presence of both quaternary ammonium cations (–N^+^ (CH_3_)_2_) and sulfonate anions (–SO_3_^−^) in Sulfobetaine molecules, their positive and negative charges can simultaneously form hydrogen bonds with the ether oxygen atoms (–O–) of Polyoxyethylene ether. Furthermore, the strong hydration layer formed by the interaction between Sulfobetaine’s sulfonate groups and Polyoxyethylene ether’s hydroxyl groups (–OH) further increases steric hindrance between molecules, thereby enhancing the system’s viscosity. Additionally, the hydrophobic alkyl chains of Polyoxyethylene ether and the hydrophobic tails of Sulfobetaine undergo hydrophobic association to form physical cross-linking points. This combination promotes the formation of micelles and larger aggregates with sizes and morphologies superior to those in single-component systems, which also contributes to the increased solution viscosity.

In general, the viscosity of a foam solution is closely related to the strength of intermolecular interactions between the functional groups of different components in the compound system. A more compact molecular arrangement usually results in greater surface viscosity. Therefore, it can be inferred that when Sulfobetaine is added at 3 wt%, the compound system achieves an optimal synergistic effect in terms of intermolecular interactions and surface viscosity enhancement between Sulfobetaine and other components. Notably, under the conditions of 7 wt% Sulfobetaine and 2 wt% Polyoxyethylene ether (Solution #4 in the table), the foam exhibits the highest viscosity. Under 6 wt% Polyoxyethylene ether and 7 wt% Sulfobetaine (Solution #14), the viscosity shows a dramatic change. In contrast, when Polyoxyethylene ether is at 8 wt% and Sulfobetaine is at 5 wt% or 7 wt% (Solutions #18 and #19), the viscosity of the foam solution reaches a minimum.

#### 3.2.2. Analysis of Wetting Time of Foam Solutions

**Figure 6:**
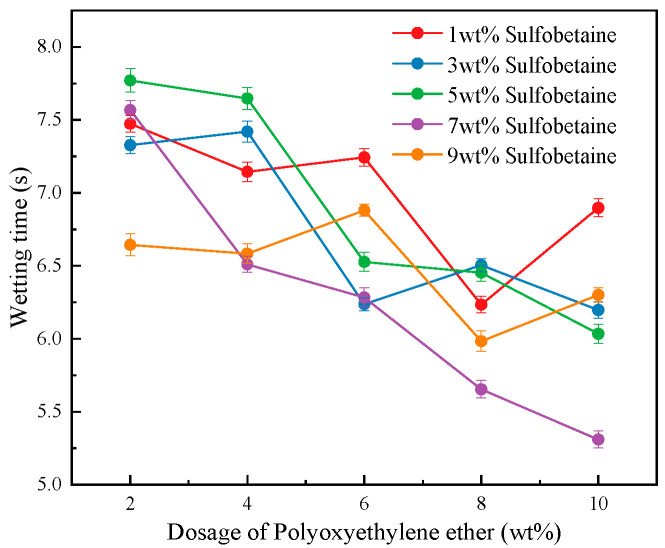
Wetting time of foam solution with the dosage of wetting agent Polyoxyethylene ether.

Figure 6 shows the trend of wetting time of foam solutions as a function of wetting agent Polyoxyethylene ether concentration under varying levels of foam stabilizer Sulfobetaine, with the concentrations of the primary and secondary foaming agents as well as the cosolvent held constant. From the figure, it can be observed that the wetting time of foam solutions generally decreases to varying degrees with increasing Polyoxyethylene ether concentration across all levels of Sulfobetaine. Specifically, at Sulfobetaine concentrations of 5 wt% and 7 wt%, the downward trend in wetting time with increasing Polyoxyethylene ether is particularly significant. Among them, the formulation with 7 wt% Sulfobetaine exhibits the best wetting performance compared to others with the same Polyoxyethylene ether content.

From a microscopic perspective, the combination of Sulfobetaine and Polyoxyethylene ether generates a positive molecular synergistic effect that significantly enhances the wettability of the foam solution. The zwitterionic nature of Sulfobetaine allows it to effectively neutralize surface charges on solids, eliminating electrostatic repulsion barriers. At the same time, its strong hydration capacity contributes to the formation of a stable hydrophilic layer at the interface. The hydrophobic chains of Polyoxyethylene ether can embed into hydrophobic micro-regions on the surface, thereby reducing interfacial energy. The ether oxygen atoms on the polyoxyethylene ether polyoxyethylene chain can form strong hydrogen bonds with the hydroxyl sulfonyl groups of sulfonamide betaine head groups, forming a dense composite interface film, which greatly improves the wetting efficiency. Moreover, the hydrophobic long chains of both surfactants tend to aggregate at the interface, forming a hydrophobic layer that effectively repels water molecules and strengthens the foam film. The flexibility of Polyoxyethylene ether’s polyoxyethylene chains also enables the molecular layer to adapt to the surface structure of solids, thereby promoting effective wetting performance.

Notably, the 5 wt% and 7 wt% Sulfobetaine concentrations correspond to relatively low viscosity levels. This suggests a potential negative feedback effect between viscosity and wetting performance, indicating that improvement in one property may inhibit the other. On the other hand, at Sulfobetaine concentrations of 3 wt% and 9 wt%—which demonstrate relatively high viscosity—the wetting time shows a fluctuating decreasing trend with increasing Polyoxyethylene ether concentration. At 1 wt% Sulfobetaine, when Polyoxyethylene ether is added at 8 wt% (Solution #16), a sharp decrease in wetting time is observed.

Therefore, in different fire suppression scenarios, depending on the type of combustible materials and combustion conditions, for situations emphasizing foam adhesion performance (such as fires with strong airflow), the addition of the foam stabilizer Sulfobetaine can be controlled at 3 wt% or 9 wt%, and the wetting agent dosage can be set at 8 wt% to enhance foam adhesion on the surface of combustible materials (Solutions #17 and #20). For cases focusing on foam wetting and coverage performance (such as fires with higher temperatures), the foam stabilizer Sulfobetaine dosage can be controlled at 7 wt%, and the wetting agent dosage at 10 wt% to achieve rapid wetting and cooling (Solution #24).

#### 3.2.3. Analysis of Surface Tension of Foam Solutions

**Figure 7:**
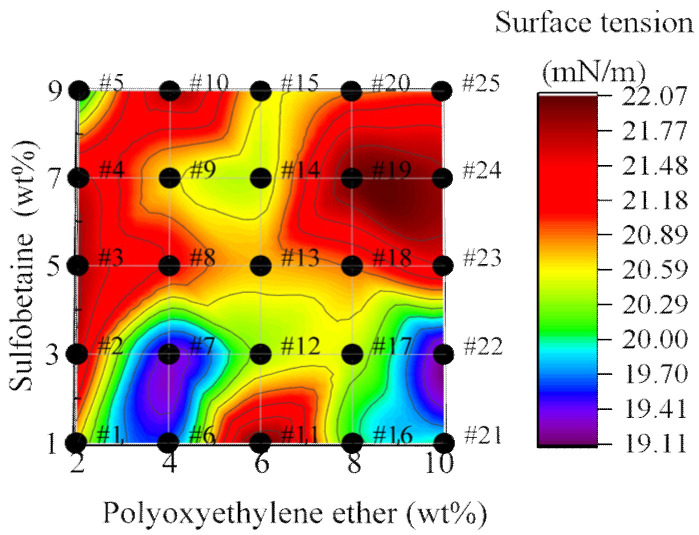
Surface Tension of Foam Solutions.

As shown in Figure 7, the surface tension test results of each group of foam solutions indicate that the surface tension is distributed relatively evenly, ranging from 19.0 to 22.50 mN/m, which meets the national standard requirement of no more than 30 mN/m. This demonstrates that the prepared foam solutions can meet practical needs and exhibit good performance.

From a microscopic perspective, the functional groups of the two can combine to form a hydrogen bond network, which not only enhances the molecular density at the interface, increasing the film density at the interface, but also reduces the surface tension of the foam solution. Additionally, the charged functional groups of Sulfobetaine’s zwitterionic structure induce dipole interactions and electrostatic shielding with the polar segments of Polyoxyethylene ether, which reduces molecular repulsion at the interface and improves the density of the adsorption layer.

From the surface tension distribution in the figure, it is evident that lower surface tensions are mainly observed in formulations with lower Sulfobetaine content. This is attributed to the thickening effect exerted by higher concentrations of Sulfobetaine. Notably, when the Sulfobetaine content is at 3 wt%, the foam solution reaches two minimum surface tension values. This aligns with the viscosity test results, indicating that at 3 wt% Sulfobetaine, the molecular interactions between Sulfobetaine and other components in the formulation reach an optimal synergistic effect.

### 3.3. Analysis of Foam Property Test Results

#### 3.3.1. Analysis of Foam Wetting Performance Test

**Figure 8:**
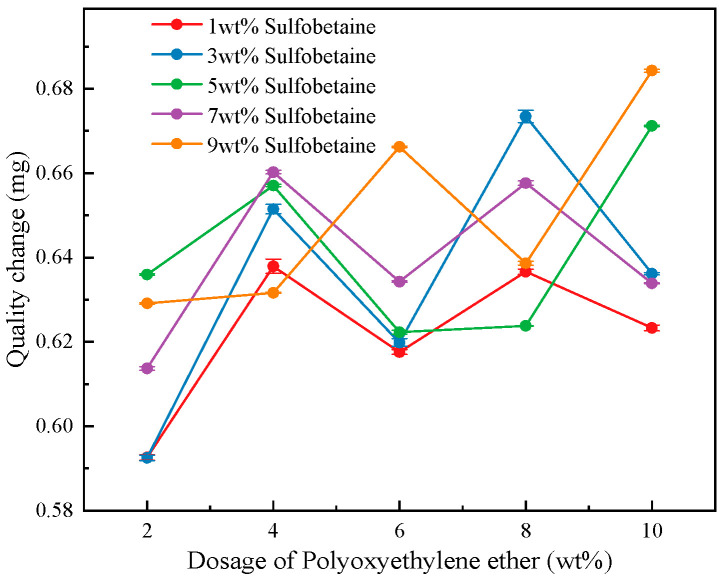
Quality change with the dosage of Polyoxyethylene ether.

As shown in Figure 8, under constant concentrations of the primary and secondary foaming agents as well as the cosolvent, the change in mass of foam-wetted cotton pieces demonstrates a varying trend with increasing concentrations of the wetting agent Polyoxyethylene ether at different levels of the foam stabilizer Sulfobetaine. It can be seen that at all Sulfobetaine concentration levels, the wetting ability of the foam on the cotton cloth first increases, then decreases, and subsequently rises again as the amount of Polyoxyethylene ether increases. Notably, favorable wetting performance is observed when the Polyoxyethylene ether concentration is at 4%, 8%, and 10%.

Furthermore, when the Polyoxyethylene ether concentration is 4%, the data curves are more centralized, suggesting good compatibility between Sulfobetaine and Polyoxyethylene ether at this level. In addition, at lower Polyoxyethylene ether concentrations (2% and 4%), the foam’s wetting ability is more significantly affected by the Sulfobetaine dosage. As the Polyoxyethylene ether concentration increases, a temporary decline in foam wetting ability is observed. This is attributed to better compatibility and synergistic effects between Sulfobetaine and Polyoxyethylene ether at lower concentrations, enhancing foam wetting performance. However, at higher concentrations, their interactions weaken, necessitating higher levels of Polyoxyethylene ether to maintain adequate wetting effectiveness.

In practical applications, considering foam loss, it is advisable to keep the Polyoxyethylene ether concentration at or above 8% to achieve optimal wetting performance. Based on the graph, several formulations with greater mass change in the cotton cloth were selected for further testing, including: Polyoxyethylene ether at 4 wt% with Sulfobetaine at 5 wt% and 7 wt% (Solutions #8 and #9), Polyoxyethylene ether at 8 wt% with Sulfobetaine at 3 wt% and 7 wt% (Solutions #17 and #19).

#### 3.3.2. Analysis of Foam Viscosity Test

**Figure 9:**
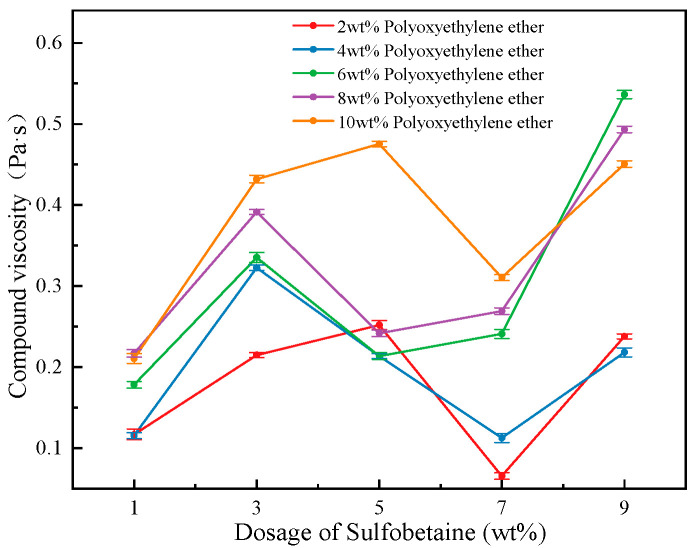
The variation in foam viscosity with the dosage of Sulfobetaine.

As shown in Figure 9, the foam composite viscosity data obtained from a rotational rheometer indicates the trend of foam viscosity as a function of Sulfobetaine stabilizer concentration under fixed concentrations of the primary and secondary foaming agents and cosolvent. Across all levels of Polyoxyethylene ether concentration, foam viscosity exhibits a trend of first increasing, then decreasing, followed by a subsequent increase as the Sulfobetaine dosage increases. Notably, excellent viscosity performance is observed at Sulfobetaine concentrations of 3 wt% and 9 wt%, consistent with the viscosity behavior of the foam solution discussed earlier.

At a low concentration of 3 wt% Sulfobetaine, the foam viscosity curve is relatively concentrated, indicating good compatibility between Sulfobetaine and Polyoxyethylene ether at this concentration, which results in favorable performance. Sulfobetaine, a commonly used surfactant, exhibits excellent foaming properties and significant thickening effects. Its zwitterionic structure partially dissociates in water, creating electrostatic repulsion and forming micelles, which contributes to an increase in viscosity. As a result, at a concentration of 9 wt%, Sulfobetaine shows excellent viscosity performance.

It is worth noting that at lower Polyoxyethylene ether concentrations (2 wt% and 4 wt%), foam viscosity remains at a relatively low level, indicating poor compatibility between Sulfobetaine and Polyoxyethylene ether under these conditions. This can be attributed to the fact that the viscosity of gel foams primarily results from compression, collision, and mutual friction between bubbles. At low wetting agent concentrations, the foam volume is small, the bubbles remain more spherical, and limited interaction occurs between them. As the wetting agent concentration increases, the number of bubbles per unit volume rises, leading to more intense collisions and, consequently, an increase in apparent viscosity.

In practical applications, the Polyoxyethylene ether concentration should be maintained at or above 6 wt% to ensure good foam adhesiveness. It can be concluded that when the Sulfobetaine concentration is 3 wt% and Polyoxyethylene ether concentration exceeds 6 wt%, the stabilizer and other components achieve optimal molecular interactions and surface thickening synergy.

#### 3.3.3. Analysis of Foam Adhesion Test

**Figure 10:**
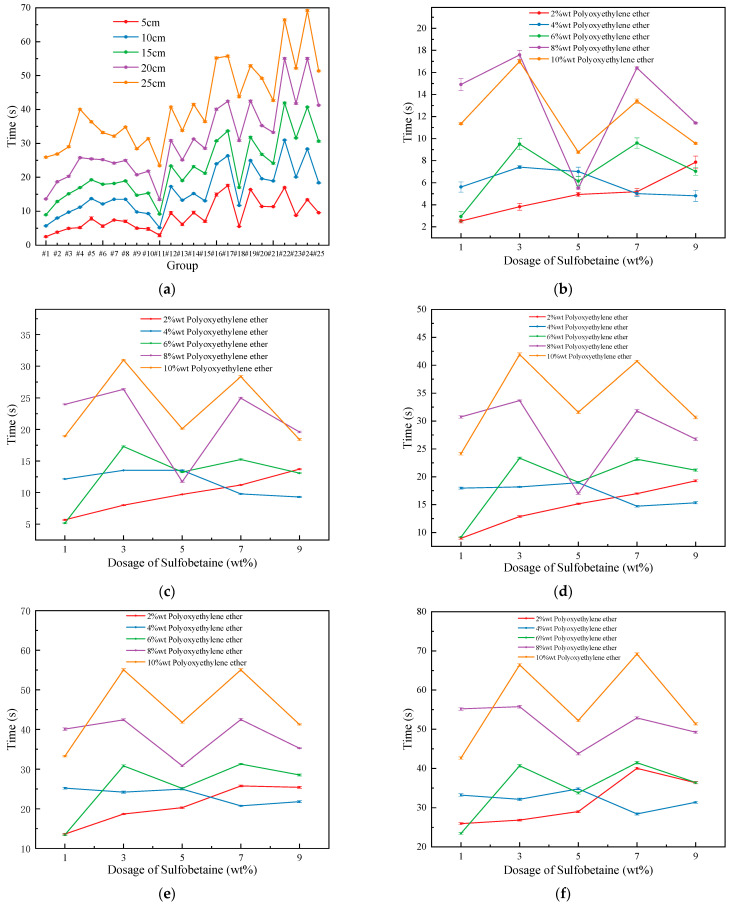
Trend of Foam Adhesion Time with the dosage of Sulfobetaine: (**a**) Overall trend; (**b**) 5 cm; (**c**) 10 cm; (**d**) 15 cm; (**e**) 20 cm; (**f**) 25 cm.

As shown in Figure 10a–f, under vertical conditions, the variation in foam adhesion time with the addition of the foam stabilizer Sulfobetaine is presented. Figure 10a illustrates that, overall, foam adhesion time across all groups follows a consistent trend with changes in vertical drop height. Figure 10b–f display the adhesion time of foams at different vertical drop heights under fixed concentrations of the primary and secondary foaming agents and the cosolvent, and at varying concentrations of the wetting agent Polyoxyethylene ether, in relation to the dosage of Sulfobetaine. Generally, at different vertical heights, foam adhesion time exhibits a similar trend—an initial increase followed by a decrease, then another increase and decrease, and finally a third increase as Sulfobetaine concentration rises.

When the concentration of polyoxyethylene ether is 2 wt%, the curve shows a continuous increasing trend due to good compatibility between the components at low concentrations, where foam adhesion time is primarily governed by the Sulfobetaine concentration. At 4 wt% Polyoxyethylene ether, the trend shifts to an increase followed by a decrease, indicating optimal molecular interaction at 3 wt% Sulfobetaine. This observation aligns with previous analysis. When the Polyoxyethylene ether concentration increases to 6 wt%, the adhesion time shows a more complex trend of increase–decrease–increase. The initial increase results from strong interaction and good adhesion, while the later rise is due to the increased Sulfobetaine concentration compensating for reduced stability of component interactions at higher wetting agent levels.

**Figure 11:**
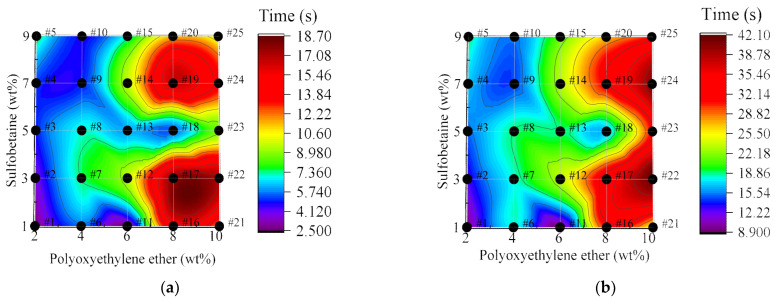
Adhesion time of foam at different falling heights: (**a**) 5 cm; (**b**) 15 cm.

As shown in Figure 11a,b, the adhesion time of foam varies at different falling heights. It can be observed that foam adhesion time differs depending on the falling distance and generally increases proportionally as the sliding distance becomes longer. Notably, at a falling height of 5 cm, the foam with 8 wt% wetting agent Polyoxyethylene ether showed a longer adhesion time. This may be due to the foam achieving an optimal level of adhesion at this concentration. As the foam slides further, some loss can occur, and a higher wetting agent concentration helps reduce this loss. As a result, at longer sliding distances, foam with 10 wt% Polyoxyethylene ether demonstrated extended adhesion time.

Moreover, it is clear that Foam #18 experienced the most significant change in adhesion time with increasing falling height. At a lower height, the foam with 8 wt% Polyoxyethylene ether even had a shorter adhesion time than the foam with 6 wt% Polyoxyethylene ether. However, with increasing falling height, the adhesion time was greatly extended. At this point, the adhesion time began to increase significantly with Polyoxyethylene ether concentration. This may be attributed to the foam’s strong wetting performance in this formulation. At short sliding distances, the foam’s performance was primarily determined by its wetting ability, making it difficult to reflect strong adhesion. As the sliding distance increased, the adhesive properties became more apparent, leading to the observed trend.

Overall, when the stabilizer Sulfobetaine is added at 3 wt% or 7 wt%, the compound system achieves strong synergistic effects in terms of intermolecular interactions and surface viscosity enhancement. Therefore, in practical applications, it is recommended to control the Sulfobetaine concentration at 3 wt% or 7 wt% and to select a relatively high concentration of Polyoxyethylene ether based on specific conditions in order to ensure optimal adhesion and effective coverage of fire sources.

**Figure 12:**
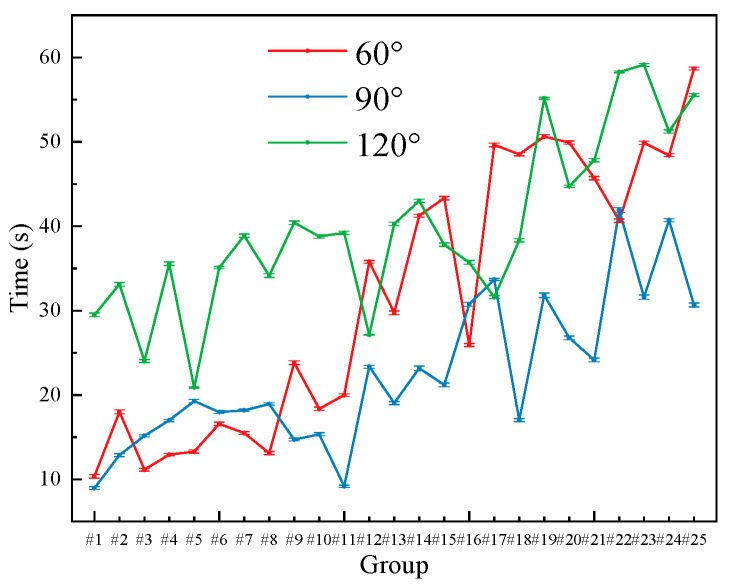
Variation in Foam Adhesion Time at Inclined Wood Surfaces.

Figure 12 shows the time required for foam to slide down from a height of 15 cm on wooden boards inclined at 60°, 90°, and 120°. Overall, under inclined conditions, foam adhesion time increases. In most cases, negative tilt angles are more effective in prolonging adhesion than positive angles. When the inclination reaches a certain level, foam may stick to the board and fail to slide off, which is consistent with real-world observations. Overall, at different vertical drop heights, the variation in foam adhesion time exhibits a consistent trend, which is consistent with the behavior of droplets falling purely under the influence of gravity.

**Figure 13:**
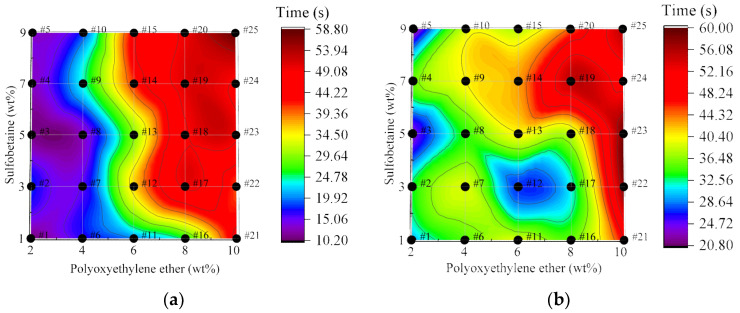
Adhesion time of foam at different inclination angles with the dosage of Sulfobetaine: (**a**) 60°; (**b**) 120°.

Figure 13a,b illustrate the variation in foam adhesion time with respect to the concentration of the foam stabilizer Sulfobetaine under different inclination angles. At an inclination angle of 60°, the variation in foam adhesion time is similar to that under vertical conditions. As the concentration of the wetting agent Polyoxyethylene ether increases, foam adhesion gradually improves. In addition, with increasing Sulfobetaine content, the adhesion time exhibits a trend of increasing first, then decreasing, followed by a second increase. Notably, when the Polyoxyethylene ether concentration reaches 8 wt%, the foam shows the best adhesion performance. However, further increases in concentration reduce the adhesion time, as excessive wetting agent content weakens the foam’s ability to adhere. At 10 wt% Polyoxyethylene ether, the foam mainly exhibits wetting behavior rather than strong adhesion.

At a 120° inclination angle, when the concentration of polyoxyethylene ether is relatively low (2 or 4 wt%), the bonding time shows a fluctuating trend of first increasing, then decreasing, and then increasing again, which is similar to the situation at 60°. However, when the Polyoxyethylene ether concentration is higher (6 or 8 wt%), the adhesion time initially decreases as Sulfobetaine content increases. This is attributed to the poor compatibility between the two components at these concentrations. The adhesion time reaches its minimum when Sulfobetaine is added at 3 wt%. As the Sulfobetaine content continues to increase, the enhanced adhesion compensates for this incompatibility, leading to a recovery in adhesion time. When the concentration of Polyoxyethylene ether reaches 10 wt%, the adhesion time increases sharply and remains at a high level. This indicates that under negative inclination angles, the concentration of the wetting agent plays a significant role in determining foam adhesion time.

### 3.4. Foam Extinguishing and Microscopic Experiments

#### 3.4.1. Analysis of Foam Extinguishing Experiments

Based on the screening results of foam solution wettability and foam adhesion, foam groups #4, #8, #9, #14, #16, #17, #19, #20, and #24 were selected for fire suppression tests. The extinguishing times for wood stack fires and the corresponding foaming ratios were recorded, as shown in Table 3.

The fire suppression process using foam on wood stacks can be divided into three stages. In the first stage, the foam interacts rapidly with the flame, absorbing heat and evaporating into steam, thereby quickly reducing the fire intensity. In the second stage, the foam acts directly on the wood surface, gradually extinguishing surface flames. In the third stage, a stable foam layer is formed, covering the surface and seeping into gaps within the wood stack, ultimately leading to complete fire suppression. All selected foam groups demonstrated good fire extinguishing performance, and none of them showed signs of reignition within ten minutes after extinguishment, meeting national standards [38].

According to the test results, foam groups #4, #14, #16, and #18 performed particularly well. However, foam group #4 exhibited excessive viscosity, resulting in a low foaming ratio, which may limit its effectiveness in large-scale practical applications. The extinguishing mechanism of the foam fire extinguishing agent developed in this study is primarily based on isolation, cooling, and suffocation. The dense foam layer effectively isolates combustible materials from air and reduces thermal radiation. Furthermore, the decomposition of the foam under heat releases liquid that contributes to cooling and evaporates to dilute the oxygen concentration in the surrounding air, achieving a suffocation effect [38,39].

For liquid fires, which are typically intense and rapidly spreading, the key to successful fire suppression lies in the foam’s ability to spread quickly across the surface and form a stable cover. Foam group #18 demonstrated excellent wettability and achieved the shortest extinguishing time, making it suitable for use in liquid fire scenarios. For common solid fires, which often involve complex fuel structures and inclined surfaces, the foam must possess strong adhesion to maintain coverage. Foam solution #19, despite its relatively low viscosity, demonstrated good adhesion in previous adhesion tests, indicating that foam adhesion is not necessarily directly related to foam solution viscosity. Foam solution #20 showed a short extinguishing time while also exhibiting good adhesion, effectively meeting practical requirements.

In conclusion, a Polyoxyethylene ether concentration of 8 wt% yielded the best overall foam performance. When the foam stabilizer Sulfobetaine is added at 5 wt%, the resulting formulation produces a wetting-type foam suitable for rapid cooling in liquid fires. When the foam stabilizer Sulfobetaine is added at 9 wt%, the foam exhibits strong adhesion, making it well suited for clinging to solid combustible surfaces.

#### 3.4.2. Analysis of Foam Microscopic Observation

Foams with better and poorer performance were selected for microscopic observation of their microstructure. The foam solutions with the longest fire suppression times (#8 and #19) and those with better fire extinguishing performance (#4, #14, #16, #18) were observed under the microscope. The following images show the changes in the foam structure over time for each group.

**Figure 14:**
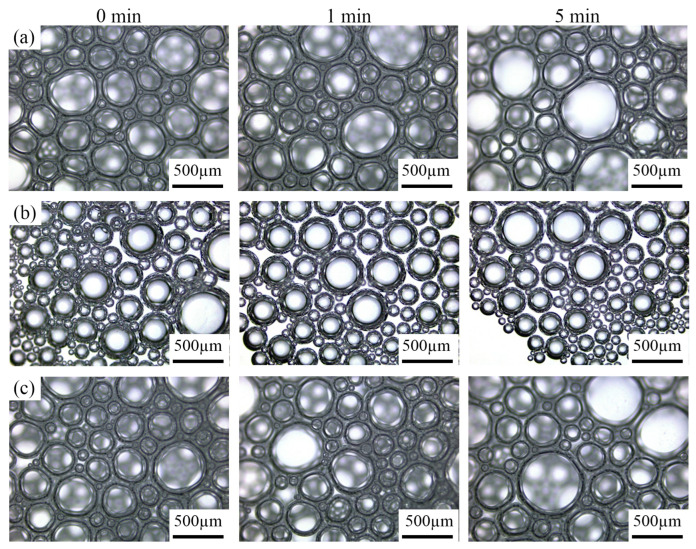

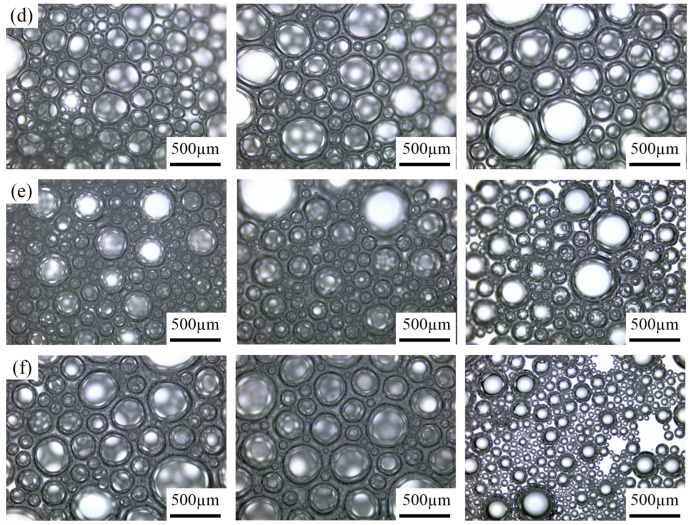
Changes in the microscopic morphology of foam: (**a**) #4; (**b**) #8; (**c**) #14; (**d**) #16; (**e**) #18; (**f**) #19.

As observed, the volume of water-based foam increases over time. This phenomenon occurs because foam is a thermodynamically unstable system. Based on the principle of energy minimization, foam tends to spontaneously reduce its interfacial area. As a result, the foam undergoes processes such as liquid film drainage, bubble coalescence, and coarsening, leading to an increase in bubble diameter [40]. From Figure 14b,f, it is clear that the foam with poor extinguishing performance loses its stability quickly and cannot form a dense foam layer, leading to inadequate foam accumulation. Figure 14a shows that although this foam formulation can form a stable foam layer, its foaming ability is relatively weak, and the generated foam volume is large.

In contrast, Figure 14c–e, which represent the best-performing foam groups, show that high foaming ratios lead to good performance. The addition of the wetting agent Polyoxyethylene ether significantly increased the thickness of the liquid film, strengthened the foam’s liquid film, and slowed down the liquid drainage rate. The addition of the stabilizer Sulfobetaine reduced the change in foam size, decreased the pressure difference between foam bubbles, and slowed down the process of bubble coalescence [1,2]. The foam with excellent fire extinguishing performance forms a stable, dense foam layer that shows a more compact distribution and higher foam volume. The foam layer changes more slowly over time, providing a stable covering effect.

## 4. Conclusions

In this study, 25 different foam concentrates were formulated based on different proportions of stabilizers and wetting agents using an orthogonal experimental method. The wetting and adhesion performance of these foam concentrates and foams were tested, and nine groups with significant performance were selected. Through foaming, fire suppression experiments, and microscopic observation, two groups of foams with the best performance were identified. The analysis of foam properties is as follows:

1. Synergistic effect of Sulfobetaine and Polyoxyethylene ether on viscosity and wettability. The combination of Sulfobetaine and Polyoxyethylene ether significantly enhances solution viscosity. The functional groups of the two surfactants interact to form hydrogen bonds and a strong hydration layer, increasing intermolecular steric hindrance. Additionally, the hydrophobic alkyl chain of Polyoxyethylene ether can associate with the hydrophobic tail of Sulfobetaine, further contributing to viscosity enhancement. The Sulfobetaine–Polyoxyethylene ether mixture also markedly improves foam wettability. Polyoxyethylene ether enables the molecular layer to conform to the solid surface topology, promoting effective wetting. The ether oxygen atoms in Polyoxyethylene ether interact strongly via hydrogen bonding with the sulfonic groups of Sulfobetaine, forming a dense composite interfacial film. The aggregation of the long hydrophobic chains at the interface generates a hydrophobic layer that effectively repels water molecules and reinforces the foam film strength.
2. Interplay between viscosity and wettability. A negative feedback effect exists between foam solution viscosity and wettability. At Sulfobetaine loadings of 3 wt% and 9 wt%, the mixture achieves optimal synergistic effects in both intermolecular interactions and surface viscosity enhancement. Wettability reaches its maximum when the concentration of Polyoxyethylene ether is maintained above 8 wt%. The surface tension of all foam solutions is relatively uniform, ranging from 19.0 to 22.5 mN/m, in accordance with national standards and adequate for practical applications.
3. Adhesiveness–wettability interaction. A similar negative feedback effect is observed between foam adhesiveness and wettability. The overall trend of foam adhesiveness follows that of the solution viscosity, with superior adhesion observed at 3 wt% and 9 wt% Sulfobetaine. Adhesion tests on inclined substrates revealed that the variation in foam adhesion time under inclined conditions is similar to that observed under vertical conditions; however, compared with the vertical orientation, the adhesion time is further extended, and foams on the underside of the board exhibit longer adhesion times than those on the upper side. Foam wettability shows minor deviations from the solution behavior but remains optimal at Polyoxyethylene ether loadings of 8–10 wt%.
4. Foam performance in fire suppression and microscopic observations. Fire-extinguishing experiments identified the optimal formulations: a wetting-type foam suitable for rapid cooling in liquid fires can be prepared with 8 wt% Polyoxyethylene ether and 5 wt% Sulfobetaine, whereas an adhesion-type foam capable of sustained attachment to solid combustible surfaces is obtained with 8 wt% Polyoxyethylene ether and 9 wt% Sulfobetaine. Microscopic observations indicate that Polyoxyethylene ether addition increases the liquid film thickness, enhances film strength, and slows drainage, while Sulfobetaine addition mitigates foam size variation, reduces pressure differences between bubbles, and delays coalescence. The foam with excellent fire suppression performance formed a stable, dense foam layer that slowly dissipated over time, providing a stable covering effect.

In future research, we will investigate the influence of environmental factors such as temperature, pH, and water quality on foam performance, and employ molecular dynamics simulations to comprehensively evaluate foam behavior at the molecular level. In addition, detailed flame dynamics monitoring and analysis will be conducted to gain deeper insights into the entire foam extinguishing process.

## Figures and Tables

**Table 1:** Foam Performance Comparison Table.

	**Foam Performance**	**Surface Tension (nN/m)**	**Expansion Ratio**	**Fire Extinguishing Time (s)**
**Foam Category**				
Wetting-type foam (this study)		20.91	8.2	12
Adhesive-type foam (this study)		20.83	7.8	20
Fluorocarbon cationic–hydrocarbon anionic surfactant foam [13]		15.33	7	28
Alkylamide propyl betaine foam [6]		17.1	7.5	114
Fluorocarbon surfactant foam containing imidazole [14]		17.5	10.5	65
6% AFFF foam extinguishing agent [15]		22.5	25.75	42
6% PF foam extinguishing agent [15]		32.5	17.78	50
Novel aqueous film-forming foam extinguishing agent [16]		16.58	9.8	65
Hydrocarbon–perfluorinated branched short-chain fluorocarbon surfactant foam [17]		19.18	11.5	31
Imidazolium short-chain fluorocarbon surfactant foam [18]		17.81	9.7	75

**Table 2:** Foam Concentrate Formulation Table.

Group	SAS	AES	Polyoxyethylene Ether	Sulfobetaine	Butyl Glycol	Deionized Water
#1	15	5	2	1	20	57
#2	15	5	2	3	20	55
#3	15	5	2	5	20	53
#4	15	5	2	7	20	51
#5	15	5	2	9	20	49
#6	15	5	4	1	20	55
#7	15	5	4	3	20	53
#8	15	5	4	5	20	51
#9	15	5	4	7	20	49
#10	15	5	4	9	20	47
#11	15	5	6	1	20	53
#12	15	5	6	3	20	51
#13	15	5	6	5	20	49
#14	15	5	6	7	20	47
#15	15	5	6	9	20	45
#16	15	5	8	1	20	51
#17	15	5	8	3	20	49
#18	15	5	8	5	20	47
#19	15	5	8	7	20	45
#20	15	5	8	9	20	43
#21	15	5	10	1	20	49
#22	15	5	10	3	20	47
#23	15	5	10	5	20	45
#24	15	5	10	7	20	43
#25	15	5	10	9	20	41

**Table 3:** Fire Suppression Test Results of Selected Foam Formulations.

Dosage	Polyoxyethylene Ether (wt%)	Sulfobetaine (wt%)	Viscosity (mPa·s)	Standard Deviation	Wetting Time (s)	Standard Deviation	Expansion Ratio	Fire Extinguishing Time (s)
#4	2	7	2.27	0.052	7.57	0.0651	4.9	13
#8	4	5	1.27	0.0458	7.65	0.0757	7.6	35
#9	4	7	1.28	0.07	6.51	0.0557	8.3	26
#14	6	7	1.28	0.017	6.28	0.0651	8	15
#16	8	1	1.67	0.02	6.23	0.0569	8.2	18
#17	8	3	1.97	0.0346	6.50	0.0451	4.9	30
#18	8	5	1.24	0.02	6.45	0.0601	8.2	12
#19	8	7	1.18	0.0346	5.65	0.0601	8.1	33
#20	8	9	1.89	0.02	5.98	0.0702	7.8	20
#24	10	7	1.16	0.0625	5.31	0.06	8.3	30

## Data Availability

The original contributions presented in this study are included in the article. Further inquiries can be directed to the corresponding author.
